# A rare case of primary malignant fibrous histiocytoma: a sarcoma of the kidney

**DOI:** 10.1186/s12894-019-0471-7

**Published:** 2019-06-04

**Authors:** Forough Ebrahimtabar, Hamid Shafi, Mohammad Ranaee, Mohammad Mehdi Darzi

**Affiliations:** 1 Faculty of Medicine, Babol University of Medical Sciences, Babol, Iran; 2 Department of Urology, Faculty of Medicine, Babol University of Medical Sciences, School of Medicine, Babol, Iran; 3 Department of Pathology, School of Medicine, Babol University of Medical Sciences, Babol, Iran

**Keywords:** Malignant fibrosis histiocytoma, Sarcoma, Kidney, Immunohistochemistry

## Abstract

**Background:**

Primary malignant fibrous histiocytoma of the kidney (MFH) is an extremely rare tumor which is indistinguishable from its’ mimics in particular renal cell carcinoma (RCC) by clinical features and preoperative imaging evaluation. Due to its high predilection to local recurrence and distant metastases, early diagnosis has great value.

**Case presentation:**

Herein, we describe a 62 year-old man with a 3-month history of abdominal discomfort and a palpable right loin mass whom computerized tomography (CT) showed a large heterogeneous solid mass in the right kidney. With the suspicious to RCC; the patient underwent radical nephrectomy. However, histopathological report revealed pleomorphic-storiform malignant fibrous histiocytoma. Immunohistochemistry study was also confirmed the diagnosis of MFH. Six month follow up showed no evidence of any recurrence.

**Conclusions:**

The therapeutic options for MFH differ from other renal tumors, hence histopathology and immunohistochemistry studies are required to establish a definite diagnosis of the disease. Despite of progress made in clinical studies and advances in diagnostic modalities, early diagnosis of MFH has not achieved yet. Further studies and accumulated experience with renal MFH are required to determine the approach to prolong survival in selected cases along with management and prognostic factors of such tumors.

## Background

Primary malignant fibrous histiocytoma (MFH) is a rare mesenchymal-derived sarcoma that belongs to a group of soft tissue malignancies with fibroblastic and histiocytic differentiation [1–3]. Although MFH is a rare disease, it has been classically defined as the most common type of renal sarcoma occurring more frequently in late adult population [4–6]. MFH is commonly found in the extremities and abdomen, but rarely in retroperitoneum with the primeval involvement of Kidney [7, 8]. Generally it is known as a poor prognostic disease with a great tendency to distant metastasis and a high rate of recurrence (> 50%) [7–11]. There are no definite clinical and radiological signs for early diagnosis of MFH [9, 12, 13]. Due to its low 5-year survival (14%) [7], early diagnostic finding can be incredibly beneficial. To the best of our knowledge, it’s the first case of renal MFH reported from Iran. Clinical manifestation of this case raised our suspicious to RCC due to its non-specified features of tumor such as hematuria that commonly seen in cases of RCC. Hereby, the clinical course of this case, histological study and management of primary renal malignant fibrous histiocytoma is discussed.

## Case report

A 62 year-old man presented with a 3-month history of obscure abdominal discomfort accompanied by sensation of mass in his right loin. He also complained from voiding difficulty and frequency. Physical examination confirmed the presence of a large, mobile and non-tender mass in the right flank. His past medical history was uneventful. Hematological tests showed leukocytosis (12.9 × 10^3^ μL) with thrombocytosis (664 × 10^3^ μL), elevated erythrocyte sedimentation rate (87 mm/hr) along with increased C-reactive protein level (86.2 mg/L). Urine analysis showed a number of RBC (15–16/hpf) in urine, explaining the microscopic hematuria. Tumor markers test revealed a raised prostate specific antigen level (PSA = 4.195 ng/ml). Serum biochemistry and chest X-ray were unremarkable. Ultrasound examination demonstrated a hypervascular encapsulated Solid cystic tumor (114 × 108 × 97 mm) in the lower zone of the right kidney. Mild hydronephrosis was seen as a result of the tumor compression. Computerized tomography (CT) also detected a large heterogeneous solid mass (131 × 129 mm) in the lower-mid portion of the right kidney with the extension to the hilum causing renal parenchymal destruction. The mass adhered to inferior vena cava (IVC) without the invasion of tumor to the IVC or thrombosis. There was no involvement of adjacent structures. In addition, a non-specific calcified nodule (12 mm) was disclosed next to the upper pole of the right kidney. The preoperative metastatic work-up showed no abnormalities. With the great suspicious to RCC, the patient underwent right nephrectomy via thoracoabdominal approach.

During hospitalization, hematological and biochemical tests were evaluated again. The levels of the erythrocyte sedimentation rate, white blood count and platelet count were all normalized. However, the laboratory data showed that creatinine level briskly increased to 4 mg/dl without oliguria. After nephrology consult and appropriate measures, the patient was discharged 7 days postoperatively in a satisfactory condition and with the following laboratory data: urea = 55.4 mg/dl, BUN = 25.9 mg/dl, creatinine = 2.25 mg/dl, glomerular filtration rate = 32 ml/min. To date, 6 months after surgery, the patient is alive with no evidence of disease recurrence.

Considering to pathological study, nephrectomy specimen was grossly 21 × 16.5 × 11 cm in size and consisted of a huge tumor attached to the kidney with the prerenal fat. The cut incision disclosed a relative hypervascular tumor with almost soft consistency arising from medial portion of upper pole to lower lobe with the extra-renal extension. The tumor was mostly separated from renal parenchyma with a definite border except some upper parts of the tumor which were admixed to the renal parenchyma. There was no evidence of adrenal gland or pericolic fat on the surface of tumor and the tumor did not invade into the perinephric tissue.

**Fig. 1:**
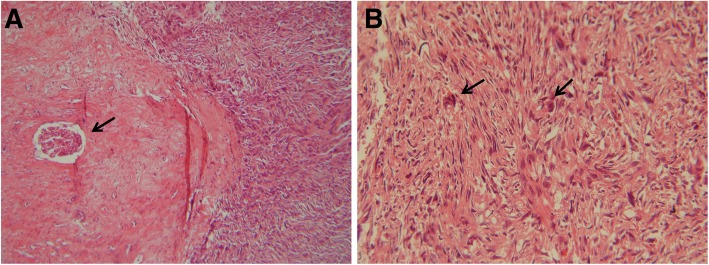
Microscopic appearance of renal sarcoma. (**a**) The tumor composed of pleomorphic spindle cells with storiform pattern which attached to kidney parenchyma (the arrow shows the glomeruli of kidney). (**b**) Frequent atypical mitosis of spindle cells has shown in this figure (haematoxylin-eosin, original magnification (**a**) 40X, (**b**) 100X)

**Fig. 2:**
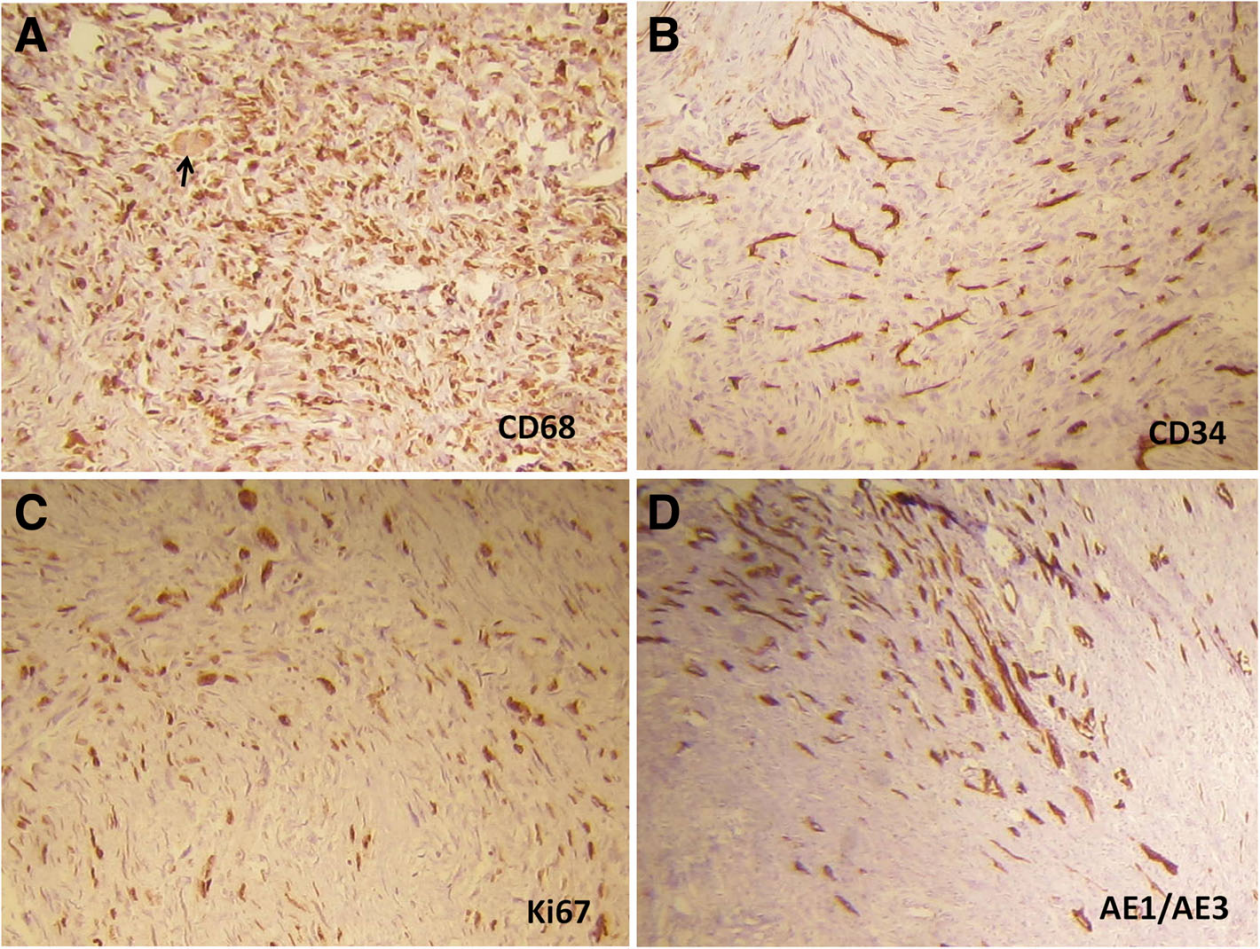
Immunohistochemistry (IHC) findings. (**a**) Tumor cells was positive for CD68 and multinucleated giant cell was observed in this Fig. (**b**) CD34 was negative in the tumor but positive in the vessel walls. (**c**) Ki67 was examined as a proliferation marker of tumor cells. (**d**) AE1/AE3 was negative in the tumor but positive in the kidney tubules. (IHC, original magnification 100X)

Microscopic examination showed a mesenchymal tumor with different patterns, including uniformed to sometimes pleomorphic nuclei spindle cells in a storiform arrangement embedded in variant amount of fibrous stroma (Fig. 1 (a)). Most part of the tumor was occupied by pleomorphic and atypical histiocytic cells arranged in a cartwheel or storiform pattern. Mononuclear to multinucleated giant cells were presented with bizarre-shaped hyperchromatic nucleus and frequent mitotic activity (Fig. 2 (a)). Areas of myxoid change and hyalinization along with aggregates of lymphocytes were also seen throughout the lesion. The tumor was not extended to pelvic, renal vessels and ureter segment. Immunohistochemical analysis revealed strong reactivity for CD68 (Fig. 2 (a)), vimentin and CD34 (Fig. 2 (b)), but the tumor was negative for CD10, Pan-cytokeratin (Fig. 2 (d)), S100 protein, desmin and H.coldesmon. Based on histological and immunohistological studies, a diagnosis of pleomorphic malignant fibrous histiocytoma was established**.**

**Table 1:** Details of survival, prognosis and other related factors of MFH cases from 2006-2017

Author	Year	Management	Chemo/radio	Pathology	immune histochemistry	Survival (months/years)	Metastasis & recurrence
Gil-Julio H et al., [9]	2012	Partial nephrectomy	------------------	pleomorphic MFH (with an abundant giant-cell population)	CD_68 +_	After a 41-month follow-up period, she was alive	During follow up period, there was no evidence of recurrence
Pathrose G et al., [16]	2015	Radical nephrectomy& splenectomy	---------	Inflammatory subtype of MFH (pleomorphic spindle cells, interspersed with foamy histiocytes)	CD_68 +_ anti α1 antichymotrysin+	-------------------------	-------------------------
Ghosh A et al., [22]	2008	Radical nephrectomy	-------------------	Inflammatory subtype of MFH (xanthoma cells with atypical histiocytes, mononuclear and multinu-Cleated giant cells)	Vimentin+	The patient died, 11 months after surgery	local recurrence, and pulmonary metastasis
Chen Y et al., [25]	2014	radical nephrectomy	The patient refused further treatment	MFH	Vimentin+, CD_68 +_	she remains alive, 78 months after first operation	9-month after surgery, a metastatic focus was found near to the abdominal aorta, however 12 and 18 months after first surgery, the metastases had disappeared (CT scan examination).
Gogus C et al., [26]	2013	Radical nephrectomy& resection of colon	Poly-chemotherapy (Adriblastina, Holoxan, Uromitexan, and radiotherapy)	MFH penetrated the capsule (Spindle-shaped fibroblastic cells in collagenous stroma)	CD10++, vimentin ++ and CD68++. α1-antitrypsin+ and α1-antichymotrypsin +	she died within the first postoperative year	Five months after operation, she was found with multiple metastases. recurrence and omental metastases were observed.
Bairwa S et al., [30]	2017	radical nephrectomy	chemotherapy	MFH (pleomorphic spindle and multinucleated polygonal cells with storiform pattern admixed with some inflammatory cells)	vimentin+	the patient died within 6 months due to recurrence.	There was evidence of recurrence (side of metastasis was not exactly mentioned).
Singh SK et al., [36]	2006	The tumors were found to be non-resectable, so biopsies only were taken.	----------------	Neutrophilic infiltrate, scattered spindle cells and foam cells	CD68+, CD34+	he died due to the disease within 1 month.	-----------------------------------------
Cormio L et al., [37]	2014	Radical nephrectomy	Adjuvant radiotherapy (50 Gy in 25 sessions)	MFH (pleomorphic, and multinucleated cells, admixed with inflammatory cells)	Vimentin+	five years after surgery, she was alive	no evidence of disease recurrence.
Hsiao PJ et al., [42]	2016	exploratory laparotomy (the tumor was not completely removed at first. 6 months later, whole tumor resected by En bloc method)	neo‑adjuvant chemotherapy with 4 cycles (mesna, doxorubicin, ifosfamide, dacarbazine).	MFH (spindle cells in a storiform pattern and polygonal or rounded cells)	vimentin+	>5 years without recurrence.	The patient survives without tumor recurrence.

## Discussion and conclusions

Renal MFH is a rarely seen mesenchymal malignant tumors comprising 1–3% of the kidney malignant tumors and about 6% of all primary renal sarcomas [12, 14, 15]. It was first established by O’Brien and Stout in 1964 with the name of malignant fibrous xanthoma [1]. MFH occurs most commonly during 6th–7th decade of life with equal sex distribution [11, 16]. The most predilection site of MFH is the extremities, followed by body trunk and retroperitoneum [7, 17]. There are also infrequent sites, such as prostate [18], spermatic cord [19], bladder [20, 21], and kidney [4, 7]. The most frequent symptoms encountered are abdominal or flank pain, palpable mass and weight loss [3, 22–24]. Although hematuria is known as a common symptom of RCC, it rarely can be an evidence of MFH [9, 24, 25]. A review of literature yielded only one case presented without any symptoms and found out incidentally via ultrasonography [24].

Nonspecific symptoms and indefinite radiological features make MFH indistinguishable from its preoperatively differential diagnosis specially RCC [3]. The diagnosis mostly depends on postoperative histology with the assistance of immunohistochemistry [24–26]. Regarding the histopatalogical studies, MFH as a mesenchymal tumor typically surrounded by a pseudocapsule. In many cases this can be found in the form of local recurrence and it’s difficult to be recognized macroscopically [27]. Specific findings suggestive of sarcoma rather than RCC include its potency to growth without lymph nodes involvement and its origination which mostly arise from capsule or perisinuous region [28]. Because renal MFH mostly arise from the capsule, hematuria can be found rarely. In our case, we found microscopic hematuria regarding to his urinalysis examination which raised our suspicious to RCC. Further studies are necessary to find out definite diagnostic and therapeutic approaches for this type of kidney sarcoma.

Histologically, MFH is divided to five types, including storiform-pleomorphic, myxoid, giant cells, inflammatory and angiomatoid [9, 29, 30], among which the storiform-pleomorphic type is the most usual and commonly high grade variant of MFH [24, 31]. MFH is characterized by typical immunepositivity for CD68, vimentin and alfa-1-antitrypsin that can differentiate it from other renal sarcomas [29] and among these markers, CD68 has become as a specific histological marker for confirming MFH [32]. Our case was also consistent with storiform arrangement of spindle cells admixed with mononuclear to multinucleated giant cells and histiocytic cells known as a histological hallmark of MFH [33] and the diagnosis was confirmed by immunohistological staining. This pleomorphic undifferentiated sarcoma portends a poor prognosis with a high risk of recurrence and distal metastases. Metastases can particularly occur in lung, lymph node, liver and bone [9, 10, 31]. Rate of metastases correlates with depth of the tumor, size, inflammatory component and myxoid change [16, 34]. Myoxid type is recognized as a high grade variant, often presented with low metastatic tendency. Therefore because of its favorable prognosis, it seems to be essential to distinct this entity from other non-myoxid types of MFH [31, 33] .

According to the literatures, most of the cases underwent radical nephrectomy as a choice of primarily treatment which is usually followed by radiotherapy, chemotherapy [12, 25, 31] and immunotherapy [35, 36]. However the success of these modalities for preventing local recurrence or distant metastases were not satisfactory, among which 25% of patients die within 1 year [7, 37]. The few reported cases of renal MFH with details are summarizedin Table 1. *Gil-Julio* et al’ reported a case with conservative treatment underwent partial nephrectomy without additional radiotherapy or chemotherapy modalities and survived after 41-month of follow-up period [9]. *Cormio* et al’ case report showed a conflicting result of a 68 year-old woman whom received adjuvant radiotherapy with considerable long-term survival consequence (72 months after surgery) as well as *Hsiao PJ* et al*’* whose patient was alive more than 5 five years after neo-adjuvant chemotherapy [37, 38]. On the other hand, *Göğüş* et al’ report revealed recurrence and metastases after five months postoperatively, and the patient died within the first postoperative year despite of polychemotherapy [26]. Therefore, the role of adjuvant radio or chemotherapy have not been found to be of obvious benefit and most of the patients succumbing to the local recurrence within the first year post radical nephrectomy [11, 25]. Our case received adjuvant chemotherapy without evidence of recurrence during 6 months follow up. Due to the poor prognosis of MFH disease and its tendency for metastasis, it is recommended that to follow up cases for longer periods.

Some case studies provide evidence of synchronization of renal MFH with transitional cell carcinoma of bladder or contralateral renal cell carcinoma [39, 40]. Therefore, it is important to be aware of other synchronous renal tumor through our work-ups.

In conclusion, malignant fibrous histiocytoma (MFH) term has implied as a rare aggressive tumor with a short term survival and poor prognosis [41, 42]. Due to the nonspecific symptoms and indefinite radiological signs, preoperative diagnosis of MFH is almost impossible which depends on postoperative histopathology. Radical nephrectomy is referred as the best choice of treatment. Adjuvant chemotherapy or radiotherapy have usually used which may not alter the poor prognosis of the disease [33]. It still needs more experience and additional case reports to early detection of the tumor related to the therapeutic consequence and prognosis.

## Abbreviations

CT: computed tomography
IVC: inferior vena cava
MFH: malignant fibrous histiocytoma
RCC: renal cell carcinoma
